# Impact of active smoking on myocardial infarction severity in reperfused ST-segment elevation myocardial infarction patients. The smoker's paradox revisited by CMR

**DOI:** 10.1186/1532-429X-17-S1-Q62

**Published:** 2015-02-03

**Authors:** Rolf Symons, Pier Giorgio Masci, Marco Francone, Piet Claus, Giovanni Donato Aquaro, Iacopo Carbone, Andrea Barison, Luciano Agati, Stefan Janssens, Jan Bogaert

**Affiliations:** 1 Imaging and Pathology, KU Leuven - University of Leuven, Leuven, Belgium; 2 Fondazione CNR/Regione Toscana, Pisa, Italy; 3 La Sapienza University, Rome, Italy; 4 Cardiovascular Diseases, KU Leuven - University of Leuven, Leuven, Belgium

## Background

Presence of intramyocardial hemorrhage (IMH) in patients with reperfused acute ST-elevation myocardial infarction (STEMI) is associated with more severe infarcts, adverse left ventricular (LV) remodeling and worse outcome. We sought to evaluate whether traditional risk factors for coronary artery disease (CAD) are related to IMH.

## Methods

In this observational, three-center study, 471 patients (60.0±11.6 years) were studied 4±1 days, and 356 patients at 4 months post-infarction (75.6%) by comprehensive by cardiovascular magnetic resonance (CMR). CAD risk factors were assessed using the most recent consensus guidelines.

## Results

IMH was present in 117 patients (24.8%) and associated with larger area at risk (AAR) and infarct size (IS), larger LV volumes, lower ejection fraction (EF) and higher troponin-I values at baseline (all p-values <0.001). At follow-up IMH was associated with lack of functional recovery and adverse LV remodeling (p<0.001). Active smoking status was associated with a younger patient age (p<0.001) and a higher prevalence of IMH, i.e., 30.9% vs 19.3% (p=0.003), while no significant association between other CAD risk factors and IMH was found. At multivariate analysis, active smoking was a strong and independent predictor of IMH (p<0.001) even after correction for LV volumes, EF, IS, AAR and myocardial salvage index. However, at follow-up, active smoking was not associated with adverse LV remodeling (defined as in increase in LV EDVi > 20%).

## Conclusions

Active cigarette smoking is clearly associated with IMH in patients experiencing a first STEMI. However, consistent with the so-called smoker's paradox, active smoking is not associated with adverse LV remodeling after 4 months.

## Funding

This study was in part funded by a FWO grant.

